# Application of various agricultural practices on sorghum forage yield and its association with water use efficiency under deficit irrigation conditions

**DOI:** 10.1038/s41598-025-32544-3

**Published:** 2026-01-08

**Authors:** Nour-El-Din Nahed, Mohamed A. Attia, Essam F. El-Hashash, Karima Mohamed El-Absy, Hassan M. El-Shaer, Ahmed M. A. Youssef, Sobhy M. A. Sallam, H. S. Khafaga, Abdelghany FI, Sara A. A. Abd-Elatty

**Affiliations:** 1 Present Address: Eco-physiology Unit, Plant Ecology and Ranges Department, Desert Research Center, P.O. Box 11753, Cairo, Egypt; 2 Plant production department, Desert Research Center, P.O. Box 11753, Cairo, Egypt; 3 Agronomy Department, Faculty of Agriculture, Al-Azhar University, Nasr City, 11651 Cairo Egypt; 4 Biology Department, University College of Tayma, University of Tabuk, P.O. Box 741, Tabuk, Saudi Arabia; 5 Animal Nutrition Department, Desert Research Center, P.O. Box 11753, Cairo, Egypt; 6 Geophysical Exploration Department, Desert Research Center, P.O. Box 11753, Cairo, Egypt; 7 Animal and Fish Production Department, Faculty of Agriculture, Alexandria University, Alexandria, Egypt; 8 Plant Genetic Resources Department, Plant Adaption Unit, Desert Research Center, P.O. Box 11753, Cairo, Egypt; 9 Plant Production Department, Faculty of Desert and Environmental Agriculture, Matrouh University, Matrouh, 51744 Egypt

**Keywords:** Environments, Planting methods, Forage yield, Water use efficiency, PCA analysis, Ecology, Ecology, Environmental sciences, Plant sciences

## Abstract

To assess the effect of various planting methods on the drought tolerance and increased forage yield and water use efficiency of sorghum, a field experiment was conducted at Ras El Hekma and Wadi El-Raml, Matrouh Governorate, Egypt, in the 2023 and 2024 growing seasons under normal irrigation and drought conditions. A drip irrigation system was used to plant sorghum. Sources of variation for seasons, locations, planting methods, and their interactions had significant effects (*P* < 0.05 or 0.01) on growth, forage yield, and water use efficiency (both fresh and dry) at most of the cuts under normal irrigation and drought conditions. Our results showed that drought stress negatively influenced growth, forage yield, and water use efficiency (in both fresh and dry) traits of sorghum at all cuts in both seasons and locations. Forage yield and water use efficiency at the various cuts are greatly influenced by the growing seasons and climate. The hole farming method significantly increased growth, forage yield and water use efficiency traits at all cuts, followed by the row method, while broadcasting resulted in the lowest values under experimental factors under study. The Wadi El-Raml location significantly boosted the traits under study at all cuts in both growing seasons under normal irrigation and drought conditions. At the sum of the three cuttings, the Wadi El-Raml location experienced improvements in fresh forage yield (6.62%), dry forage yield (8.04%), fresh water use efficiency (6.56%), and dry water use efficiency (7.18%) under drought conditions, in comparison to the Ras El Hekma location. There are positive associations among all studied traits at all cuts under normal irrigation and drought conditions. The significance of planting methods and locations in both growing seasons as the primary contributing traits for sorghum’s water use efficiency and fodder yield under drought conditions was shown using STI and PCA based on the phenotypic correlation. Therefore, to increase the forage output and water use efficiency of sorghum, it is highly advised that the hole farming method be applied to the Wadi El-Raml area in Egypt under drought conditions.

## Introduction

A C4 crop with a high energy content, sorghum (*Sorghum bicolor* L. Moench) is distinguished by its short growth cycle, high biomass output, and resilience to stress^1^. Sorghum is a multipurpose annual plant species. It is a good source of sugar, grain, and fodder. Sorghum can be grown for fodder and as broomcorn in addition to being a grain that is regarded as the fifth most important crop after wheat, rice, maize, and barley^2,3^. When compared to other crops in the cereal family, sorghum is a serious crop that can withstand drought well^4^. Although sorghum is a staple crop in dry and semi-arid areas of the world, water stress frequently affects it before and after blooming, which can drastically impair grain output^5^. Though there are notable differences between their methods of use, sorghum and maize are comparable in terms of feed quality and commercial worth. Although sorghum can be eaten in a variety of ways, including silage, wet forage (high moisture green fodder), dry fodder (hay), and direct grazing as pasture or after livestock collect it, silage is the most common way that maize is eaten^6^. Additionally, compared to maize, sorghum is a more economically advantageous crop due to its potential to be harvested as a crop with many cuttings^7^. Sorghum’s ability to readily adapt to a variety of agroecological situations is one of its most significant benefits. It is a significant crop for bioenergy and a great supplement to ruminant diets. Due to its great degree of drought and temperature tolerance, forage sorghum can provide large amounts of food with fewer resources^8^. Recent statistics show that sorghum was cultivated on almost 40.73 million hectares worldwide in 2025/2026, with 0.15 million hectares in Egypt. Egypt had a high potential yield, with an average yield of more than 5.20 metric tons ha^-1^ compared with a worldwide 1.54 metric tons/ha^9^.

The substantial discrepancy between the actual average yield that farmers acquire on their fields and the prospective or attainable fodder yield (what can be reached with the optimum agronomic practices and appropriate varieties) is known as the sorghum fodder yield gap. This disparity is frequently significant, suggesting a sizable chance to boost output. Assefa et al.^10^ shown that despite an increase in yield potential brought about by advancements in agronomy or hybrid creation, there is a significant yield gap in sorghum and a significant year-to-year variance in yields. Potential and achievable on-farm water-limited yields differ significantly^11^. The actual yield (based on USDA statistics) and the projected non-irrigated yield were found to differ by 66 to 96%. Timely planting and optimal maintenance of study plots, which are challenging for producers to reproduce when farming a vast region, are partly responsible for this yield disparity^10^. With an average global actual yield of 1.4 to 2.5 tonnes per hectare (t/ha) compared to theoretical yields that can reach over 12 t/ha under ideal circumstances, the global yield gap for sorghum is significant and extremely varied by area^9^. Evidence regarding the contribution of cultivar-specific characteristics to yield stability under varying soil water retention capacities, such as plant height, tillering capability, and dry matter content, is still lacking. In areas where climate unpredictability is becoming more prevalent, closing this gap is essential to maximizing sorghum production as a sustainable fodder supply^12^. Significant yield variability in rainfed environments is caused by agronomic practices, water scarcity, and irregular rainfall patterns, particularly during crucial growth times.

Crop stability and productivity are seriously threatened by climate variability, especially by changing precipitation and warming temperatures^13^. Plant growth requires water, and one of the biggest obstacles to crop productivity is drought^14^. A common abiotic stressor that restricts crop development and productivity, drought has a major effect on ecosystems, agriculture, and food security^15^. According to recent research, crop physiology activities are significantly hampered by drought stress at several developmental growth phases. This delays maturation and results in significant production losses^16^. One important factor is drought. A key tactic for maintaining increased yields is improving drought resistance, with an emphasis on creating cultivars that can withstand drought^17^. Numerous elements of plant growth, including as plant height, fresh and dry weight, leaf number, leaf area, root length, and number of root systems, are suppressed by drought stress, according to earlier research^17–19^.

One well-known crop type that can withstand drought is sorghum, and sorghum landraces have unique alleles that let them adapt^14^. In areas vulnerable to climatic stress, sorghum is a promising crop for future food and feed security because of its resilience and drought tolerance^13^. Sorghum’s diploid genomic structure and effective photosynthetic system make it a great crop that can withstand drought^20^. According to Tsehaye et al.^5^, different genotypes of sorghum react differently to drought stressors before and after blooming.

Agronomic techniques like planting and irrigation have a big influence on plant yields. For optimal crop production, planting procedures should be modified based on soil types and climate changes. The crop can thrive in later stages of crop growth and development when the right planting technique is used^21,22^. Insufficient land availability, improper timing and techniques for sowing, a shortage of high-quality forage seeds, poor irrigation and nutrition management, weed infestation, insufficient plant protection, etc. are the primary reasons for low forage production^23^. The production and quality of biomass are significantly impacted by management factors, such as planting techniques and the cultivation of appropriate cultivars. The final grain and biomass yield of maize and sorghum are impacted by improper sowing techniques such as broadcast and flat sowing, which lead to poor germination and stand establishment^24^. Consequently, compared to traditional sowing techniques, improved planting techniques such as ridge and bed sowing enhanced seed germination and assisted plants in making better use of light, land, and other inputs^25^. Additionally, because of the proper soil conditions, ridge and raised beds enhance root growth, which significantly boosts water and nutrient uptake and increases maize biomass output^26^. Compared to traditional broadcasting and line sowing, ride and bed sowing significantly boosted the grain and dry matter productivity of maize^27^. While the furrow serves as a planting belt, collects precipitation that falls directly on the surface belt, and receives runoff from the ridges, ridges are crucial for collecting rainwater^28^. The crop’s microenvironment can be directly impacted by different planting techniques, which can then affect the crop’s growth characteristics and yield components^29^. The previous literature reviews present clear hypotheses regarding the importance of this study in addressing the problem of water scarcity.

Since these procedures are required to increase forage yield and water use efficiency, it is essential to comprehend the complex interactions between environmental factors and planting techniques under normal irrigation and drought conditions to maximize sorghum forage yield. Therefore, under normal irrigation and drought conditions, the current field experiments were conducted to examine the effects of seasons, locations, and planting techniques (drill in row, broadcasting, and hole farming) on the growth, forage yield and water use efficiency traits (in both fresh and dry) of sorghum in Egypt.

## Materials and methods

### Experimental design and treatment details

Seeds of the Sorghum Hendy variety (Hybrid Sorghum Mecca) were imported from India. Under normal irrigation and drought conditions, separate field experiments have been carried out in the 2023 and 2024 growing seasons at the two private farms in Ras El Hikma at East of Marsa Matrouh City (Latitude 27°52 × 29.6” N and Longitude 31°05 × 21.4” E) and Wadi El-Raml at West of Marsa Matrouh City (Latitude 27°09 × 13.1” N and Longitude 31°16 × 17.8” E), Matrouh Governorate, Egypt. Identifying the best planting methods (hole farming, drill in row, and broadcasting) for the Sorghum Hendy variety in terms of growth, forage yield, and water usage efficiency attributes in both growing seasons and locations under normal and drought conditions was the aim of this study. In each experiment, sorghum seeds were planted under three planting methods in a randomized complete block design with three replications. Each experimental unit measured 15 by 40 (400 m^2^), with a row spaced 20 cm apart and a hole spaced 10 cm apart. Each subplot included two border rows and a buffer gap of 2 m between neighboring plots to reduce treatment overlaps and positional bias. Planting took place on May 15 and 20 of the 2023 and 2024 growing seasons, respectively. Each fed had a seed rate of 20 kg. A drip irrigation system was used to plant sorghum. During the aforementioned sowing dates, sorghum seeds were planted in hills (about five seeds per hill) in the two furrow ridges, covered with sand, and then immediately irrigated. The normal and drought experiments for each season and location were irrigated with water amounts of 2200 and 2000 m^3^ per fed, respectively. To reduce environmental fluctuations as much as possible, all agronomic procedures were followed, and the crop was sown in a single day under consistent field conditions. The first, second, and third cuts were taken after 50, 180, and 105 days after the sowing date, respectively. Using established procedures by Page et al.^30^, Table 1 displays the findings of soil analysis for 0–30 cm depth prior to planting at Ras El Hekma and Wadi El-Raml sites, Matrouh Governorate, Egypt, in two growing seasons (2023 and 2024).

**Table 1:** Some chemical properties of soil samples of the surveyed sites.

Sites	Depth	pH	Ec (dS/m)	Ca (Meq/l)	Mg (Meq/l)	Na (Meq/l)	K (Meq/l)	CO_3_ (Meq/l)	HCO_3_ (Meq/l)	Cl (Meq/l)	SO4 (Meq/l)	SAR
Ras El Hekma	0–30	8.1	0.88	1.52	1.33	5.32	0.45	0	4.70	2.23	1.79	4.45
	30–60	8.1	0.85	1.55	1.55	5.01	0.58	0	4.34	1.73	2.34	4.03
Wadi El-Raml	0–30	7.8	10.9	41.73	29.44	58.42	0.90	0	6.70	119.15	7.02	9.79
	30–60	7.8	4.75	17.26	6.48	21.89	0.82	0	4.37	32.07	10.91	6.35

## Irrigation water applied (IWA)

The daily reference evapotranspiration ($\:{ET}_{o}$) values were estimated based on FAO Penman-Monteith method using the latest five-year average of weather data from the meteorological station at two locations, where our experiment was conducted^31^. The crop water requirements expressed as crop evapotranspiration ($\:{ET}_{c}$; mm d^− 1^) according to the Allen et al.^31^ equation was calculated as follows:

$$\:{ET}_{c}={ET}_{o}x{K}_{c}$$

where, $\:{ET}_{o}$ and $\:{K}_{c}$, denotes evapotranspiration (mm d^− 1^) and crop coefficient value, respectively, which differs from one growth stage to another as described by Allen et al.^31^ and Brouwer and Heibloem^32^. The amount of IWA per experimental plot during the irrigation regime was computed following the equation given by Allen et al.^31^ as follows:

$$\:IWA\left({m}^{2}\right)=\frac{{ET}_{c}xAx{I}_{i}}{Eax1000x(1-LR)}$$

where $\:{ET}_{c}$, A,$\:\:{I}_{i}$, Ea, and LR, respectively, are the crop water requirements (mm d^− 1^), experimental plot area (m^2^), irrigation intervals (d), efficiency of irrigation system, which was considered 0.6, and leaching water requirements.

Using one PVC (polyvinyl chloride) pipe (50 mm diameter × 1 m length) for each plot, the IWA was transferred to cover the whole plot surface area. The irrigation water quota transferring across each PVC pipe for each plot was calculated following the equation given by Israelsen and Hansen^33^ as follows:

$$\:Q=\frac{CA\sqrt{2gh}}{1000}$$

where Q, C, A, g, and h, are the irrigation water discharge (l s^− 1^), discharge coefficient, PVC pipe’s cross section area (cm^2^), gravity acceleration (cm s^− 2^), effective head of water (cm) over the center of piper making flow free, respectively. A guard border of 2 m width between the adjacent experimental plots was in each replication to avoid the border effects. Likewise, another one with 5 m width as a separator under two irrigation treatments (2200 and 2000 m^3^ fed^− 1^) was maintained to avoid water infiltration from one to another treatment.

## Measurements of studied traits

To determine the plant height (cm), number of tillers/m^2^, fresh weight (ton/fed), and dry weight (ton/fed) under normal irrigation and drought circumstances, one square meter was randomly selected from each plot, and the two cuts were taken after the first. Every cut was picked by hand. Once the fresh weight of the complete sample was recorded in the field, two kilograms were taken as a subsample and divided into leaf and stem. The dry weight of the plant components was measured following three days of oven drying at 80 °C. Fresh and dry water use efficiencies (kg/m³) were calculated by dividing the fresh and dry forage yield (kg/fed) by the amount of water received in both growing seasons (m³/fed), respectively^34^, according to the following equations:

$$\:Fresh\:water\:use\:efficiency=\frac{Fresh\:forage\:yield\:(kg/fed)}{Wster\:volume\:({m}^{3}/fed)}$$

$$\:Dry\:water\:use\:efficiency=\frac{Dry\:forage\:yield\:(kg/fed)}{Wster\:volume\:({m}^{3}/fed)}$$

### Statistical analysis

The assumptions of normality and homogeneity of variances were verified before performing the analysis. A three-way ANOVA test and the coefficient of variation (CV%) were applied to the measured data in accordance with Steel and Torrie^35^ approach in order to detect any notable differences in the influence of the experimental components and their interactions. The means at *p* < 0.05 were compared using the least significant difference (LSD) test. Small letters were used to compare the column means. Gomes^36^ states that the CV% estimates were separated into four groups: low (CV < 10%), moderate (10%< CV ≤ 20%), high (15.0% ≤ CV ≤ 21.0%), and extremely high (CV ≥ 21%). Fernandez^37^ method was used to calculate the stress tolerance index (STI) for fresh and dry forage yields across the experimental factors under normal $\:{(Y}_{p})$and drought $\:{(Y}_{s})\:$conditions, as follows: $\:STI={(Y}_{p}\times\:{Y}_{s})/{\left({\stackrel{-}{Y}}_{p}\right)}^{2},$ where $\:{\stackrel{-}{Y}}_{p}$ indicates the mean of all factors in normal conditions. To further understand the relationship between the experimental treatments and the qualities being studied, principal component analysis (PCA) was employed. The ANOVA and PCA were performed using SPSS version 20 and OriginPro 2025b 10.2.5.212, respectively.

## Results

### Seasonal weather

**Fig. 1:**
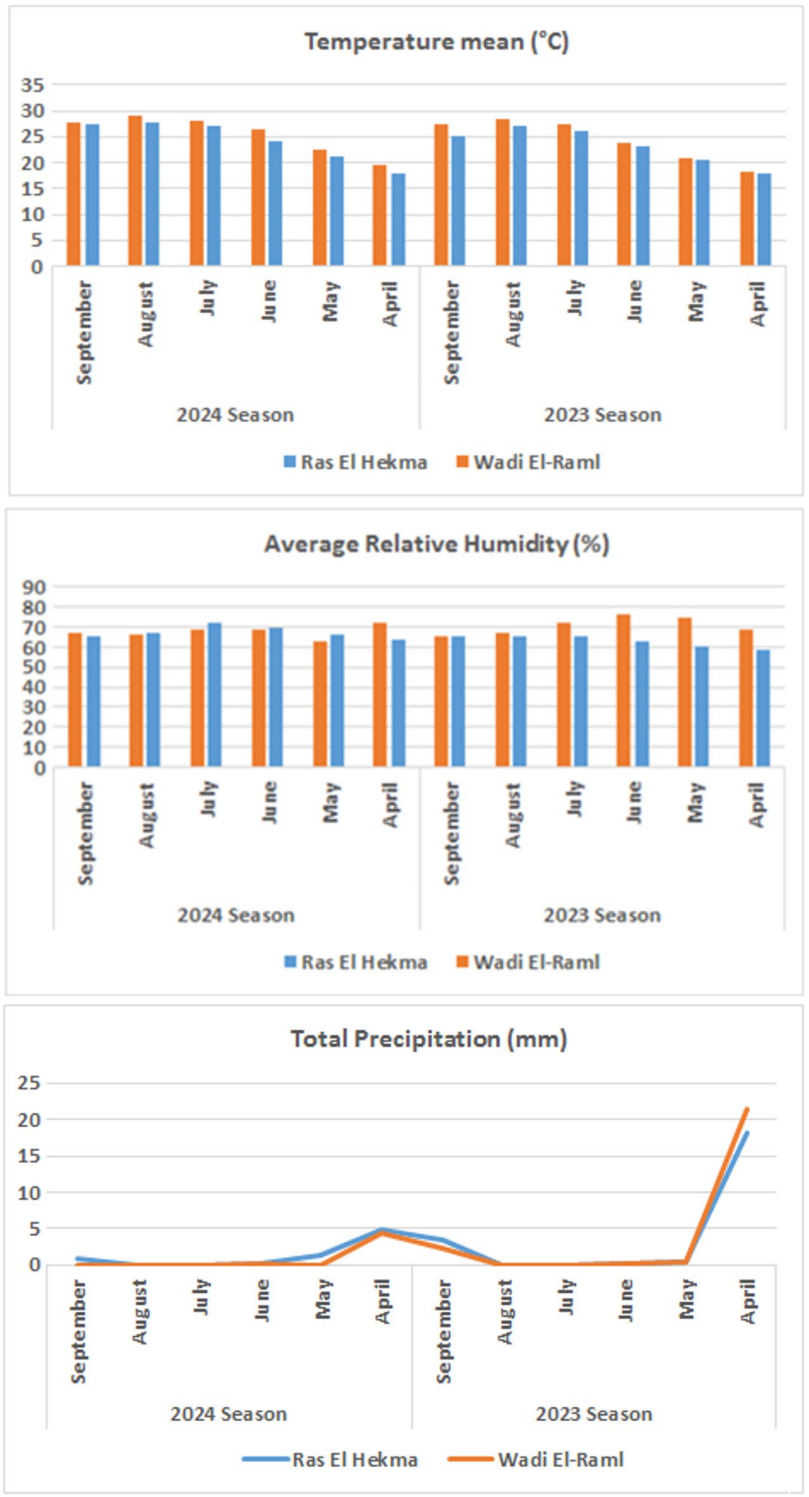
Climatic data at Ras El Hekma and Wadi El-Raml locations, Matrouh Governorate, Egypt, in the 2023 and 2024 growing seasons.

Figure 1 displays climate data from April to September across the two growing seasons and locations. Differences in mean temperature, total precipitation, and average relative humidity resulted in significant variations between the two years and between the locations under study. Furthermore, we noted severe occurrences of unevenly distributed rainfall in both seasons and locations, as well as significantly higher mean temperatures and average relative humidity during the 2024 growing seasons and at the Wadi El-Raml location under study. Both growing seasons also had higher average temperatures, total precipitation, and average relative humidity in the Wadi El-Raml location compared to the Ras El Hekma location. August had higher temperatures for both growth seasons and locales. April saw lower temperatures in both growing seasons and regions. The highest average relative humidity was noticed for July at the Ras El Hekma location in both growing seasons, and for June and April at the Wadi El-Raml location during the 2023 and 2024 growing seasons, respectively. The lowest average relative humidity was observed for April at the Ras El Hekma location in both growing seasons, and for September and May at the Wadi El-Raml location during the 2023 and 2024 growing seasons, respectively. In both growth seasons and localities, April had the greatest total precipitation. At both locations, overall precipitation during the 2023 growing season was higher than during the 2024 growing season.

## Analysis of variance (ANOVA)

The ANOVA data for plant height, No. of tillers, forage yield (both fresh and dry), and water use efficiency (both fresh and dry) at all cuts under normal irrigation and drought conditions are presented in Table 2. The ANOVA revealed significant variation (*P* < 0.05 or 0.01) between the main effects of seasons and locations for plant height at the first, third, and mean cuts under normal conditions and at the first and mean cuts under drought conditions; and for the number of tillers, fresh forage yield, dry forage yield, fresh water use efficiency, and dry water use efficiency traits at all cuts in both conditions. The location factor had a highly significant variation for plant height at the third cut under drought conditions. The study showed significant differences (*P* < 0.05 or 0.01) in the planting methods for all studied traits at all cuts in both conditions, except plant height at the second cut and fresh forage yield and fresh water use efficiency at the first cut under drought conditions, which had insignificant differences.

The interaction of seasons x locations was significant (*p* < 0.05 or 0.01) under normal and drought conditions for plant height at the first, third, and mean cuts, and for fresh forage yield, dry forage yield, fresh water use efficiency, and dry water use efficiency at the second and third cuts. While number of tillers/m^2^ had an insignificant difference at all cuts under normal and drought conditions. The effects of the season x planting methods interaction were significant (*p* < 0.05 or 0.01) for plant height at the third cut under normal conditions and at the third and mean cuts under drought conditions; for dry forage yield and dry water use efficiency at the second and third cuts under normal conditions and at the first, third and, mean cuts under drought conditions; and for dry water use efficiency at the total of the three cuts under drought conditions. At the same time, the number of tillers/m^2^ and fresh forage yield had an insignificant difference at all cuts under normal and drought conditions. The interaction between locations and planting methods was significant (*p* < 0.05 or 0.01) for No. of Tillers\m2 at the second cut under normal and drought conditions; for fresh forage yield and Fresh water use efficiency at the third cut under normal conditions; and for dry forage yield and dry water use efficiency at the second and third cuts under normal conditions and at all cuts except the second cut under drought conditions. In contrast, plant height showed no significant difference at all cuts under both normal and drought conditions. Significant interactions among seasons, locations, and planting methods were observed on plant height at mean cut under normal conditions and at the third and mean cuts under drought conditions; on fresh forage yield and fresh water use efficiency at the third cut under normal conditions; on dry forage yield and dry water use efficiency at the second and third cuts under normal conditions and all cuts under drought conditions except the second cut. In comparison, the number of tillers/m^2^ had an insignificant difference at all cuts under normal and drought conditions.

Low coefficients of variation (CV%) were noticed for all studied traits at all cuts, which were less than 10%, except for plant height at the second cut during normal (17.73%) and drought (24.68%) conditions, for fresh forage yield and fresh water use efficiency at the third cut (10.09%) under drought conditions, and for dry forage yield and dry water use efficiency at the second (19.55%) and third (12.00%) cuts under drought conditions. The lowest CV% value (1.62%) was found for the plant height at the mean of the three cuttings.

**Table 2:** ANOVA test (p-values) for the effect of the seasons, locations, and planting methods on studied traits of sorghum at the three cuts as well as the mean and total of the three cuts under normal irrigation and drought conditions.

S.O.V	Normal conditions				Drought conditions			
	1st cut	2nd cut	3rd cut	Mean	1st cut	2nd cut	3rd cut	Mean
Plant height (cm)								
Seasons (S)	0.04^*^	0.46	0.00^**^	0.00^**^	0.00^**^	0.41	0.25	0.00^**^
Locations (L)	0.00^**^	0.72	0.00^**^	0.00^**^	0.00^**^	0.88	0.00^**^	0.00^**^
S x L	0.01^*^	0.64	0.02^*^	0.00^**^	0.00^**^	0.14	0.00^**^	0.00^**^
Planting methods (P)	0.00^**^	0.03^*^	0.00^**^	0.00^**^	0.00^**^	0.24	0.00^**^	0.00^**^
S x P	0.61	0.60	0.00^**^	0.95	0.69	0.53	0.00^**^	0.00**
L x P	0.91	0.52	0.86	0.27	0.92	0.27	0.68	0.40
S x L x P	0.76	0.23	0.18	0.01^*^	0.98	0.56	0.02^*^	0.01^*^
C.V.%	3.21	17.73	1.69	1.62	2.29	24.68	2.59	1.97
No. of Tillers\m2								
Seasons (S)	0.03^*^	0.00^**^	0.00^**^	0.00^**^	0.02^*^	0.00^**^	0.00^**^	0.00^**^
Locations (L)	0.10	0.00^**^	0.00^**^	0.00^**^	0.00^**^	0.00^**^	0.00^**^	0.00^**^
S x L	0.94	0.52	0.37	0.45	0.71	0.21	0.66	0.71
Planting methods (P)	0.00^**^	0.00^**^	0.00^**^	0.00^**^	0.00^**^	0.00^**^	0.00^**^	0.00^**^
S x P	0.98	0.43	0.96	0.75	0.83	0.46	0.50	0.62
L x P	0.22	0.00^**^	0.51	0.31	0.48	0.00^**^	0.22	0.22
S x L x P	1.00	0.52	0.89	0.66	0.78	0.99	0.64	0.89
C.V.%	8.73	6.08	7.46	3.99	7.76	6.67	3.99	4.44

S.O.V	Normal conditions					Drought conditions				
	1st cut	2nd cut	3rd cut	Mean	Sum	1st cut	2nd cut	3rd cut	Mean	Sum
Fresh forage yield (t/fed)										
Seasons (S)	0.02^*^	0.00^**^	0.00^**^	0.00^**^	0.00^**^	0.03^*^	0.00^**^	0.00^**^	0.00^**^	0.00^**^
Locations (L)	0.00^**^	0.00^**^	0.00^**^	0.00^**^	0.00^**^	0.00^**^	0.00^**^	0.00^**^	0.00^**^	0.00^**^
S x L	0.72	0.00^**^	0.00^**^	0.53	0.53	0.82	0.00^**^	0.00^**^	0.50	0.50
Planting methods (P)	0.00^**^	0.00^**^	0.00^**^	0.00^**^	0.00^**^	0.23	0.00^**^	0.00^**^	0.00^**^	0.00^**^
S x P	0.95	0.18	0.13	0.70	0.70	0.78	0.13	0.99	0.48	0.48
L x P	0.92	0.66	0.08^*^	0.36	0.36	0.75	0.16	0.84	0.64	0.64
S x L x P	0.91	0.35	0.05^*^	0.71	0.71	0.67	0.26	0.91	0.77	0.77
C.V.%	7.39	5.49	7.82	3.64	3.64	9.23	8.53	10.09	6.08	6.08
Dry forage yield (t/fed)										
Seasons (S)	0.04^*^	0.00^**^	0.00^**^	0.00^**^	0.00^**^	0.00^**^	0.00^**^	0.00^**^	0.00^**^	0.00^**^
Locations (L)	0.00^**^	0.00^**^	0.00^**^	0.00^**^	0.00^**^	0.00^**^	0.00^**^	0.00^**^	0.00^**^	0.00^**^
S x L	0.88	0.00^**^	0.00^**^	0.26	0.26	0.11	0.00^**^	0.00^**^	0.24	0.89
Planting methods (P)	0.00^**^	0.00^**^	0.00^**^	0.00^**^	0.00^**^	0.00^**^	0.02^*^	0.00^**^	0.00^**^	0.00^**^
S x P	0.96	0.01^*^	0.00^**^	0.67	0.67	0.02^*^	0.35	0.00^**^	0.00^**^	0.14
L x P	0.85	0.01^*^	0.00^**^	0.52	0.52	0.01^*^	0.34	0.00^**^	0.00^**^	0.06^*^
S x L x P	0.99	0.02^*^	0.00^**^	0.92	0.92	0.04^*^	0.77	0.00^**^	0.00^**^	0.01^*^
C.V.%	8.02	4.93	6.79	4.67	4.67	6.68	19.55	12.00	4.61	6.38
Fresh water use efficiency (kg/m^3^)										
Seasons (S)	0.02^*^	0.00^**^	0.00^**^	0.00^**^	0.00^**^	0.03^*^	0.00^**^	0.00^**^	0.00^**^	0.00^**^
Locations (L)	0.00^**^	0.00^**^	0.00^**^	0.00^**^	0.00^**^	0.00^**^	0.00^**^	0.00^**^	0.00^**^	0.00^**^
S x L	0.72	0.00^**^	0.00^**^	0.53	0.53	0.82	0.00^**^	0.00^**^	0.50	0.50
Planting methods (P)	0.00^**^	0.00^**^	0.00^**^	0.00^**^	0.00^**^	0.23	0.00^**^	0.00^**^	0.00^**^	0.00^**^
S x P	0.95	0.18	0.13	0.70	0.70	0.78	0.13	0.99	0.48	0.48
L x P	0.92	0.66	0.08^*^	0.36	0.36	0.75	0.16	0.84	0.64	0.64
S x L x P	0.91	0.35	0.05^*^	0.71	0.71	0.67	0.26	0.91	0.77	0.77
C.V.%	7.39	5.49	7.82	3.64	3.64	9.23	8.53	10.09	6.08	6.08
Dry water use efficiency (kg/m3)										
Seasons (S)	0.04^*^	0.00^**^	0.00^**^	0.00^**^	0.00^**^	0.00^**^	0.00^**^	0.00^**^	0.00^**^	0.00^**^
Locations (L)	0.00^**^	0.00^**^	0.00^**^	0.00^**^	0.00^**^	0.00^**^	0.00^**^	0.00^**^	0.00^**^	0.00^**^
S x L	0.88	0.00^**^	0.00^**^	0.26	0.26	0.11	0.00^**^	0.00^**^	0.23	0.23
Planting methods (P)	0.00^**^	0.00^**^	0.00^**^	0.00^**^	0.00^**^	0.00^**^	0.00^**^	0.00^**^	0.00^**^	0.00^**^
S x P	0.96	0.01^*^	0.00^**^	0.67	0.67	0.02^*^	0.50	0.00^**^	0.00^**^	0.00^**^
L x P	0.85	0.01^*^	0.00^**^	0.52	0.52	0.01^*^	0.85	0.00^**^	0.00^**^	0.00^**^
S x L x P	0.99	0.02^*^	0.00^**^	0.92	0.92	0.04^*^	0.35	0.00^**^	0.00^**^	0.00^**^
C.V.%	8.02	4.93	6.79	4.67	4.67	6.68	19.55	12.00	4.61	6.38

Statistically significant differences at *p ≤ 0.05 and **p ≤ 0.01; ns: indicate the non-significant difference.

## The main effects of experimental factors

The main effects of seasons, locations, and planting methods at all cuts for all studied traits under normal irrigation and drought conditions are presented in Table 3. The highest plant height and No. of tillers/m^2^ were obtained in 2023 growing seasons at all cuts in both conditions, while the highest values for forage yield and water use efficiency (both fresh and dry) traits were observed with 2024 growing seasons at all cuts, except the second cut in both conditions. As for studied locations, all studied traits at all cuts in both conditions increased in Wadi El-Raml than in Ras El Hekma, except No. of tillers/m^2^ at the first cut and forage yield and water use efficiency (both fresh and dry) at the second cut. Compared with other planting methods, the hole farming method enhanced all the studied traits at all cuts under normal irrigation and drought conditions. The hole farming method produced the highest forage yield and water use efficiency of sorghum, followed by drill-in-row and broadcasting methods at the three cuts, as well as the mean and total of the three cuttings in both conditions. Under every planting method in both seasons and locations, every attribute that was examined at the first cut performed better than the second and third cuts in both conditions. Drought conditions reduced all studied traits at all cuts compared with the normal irrigation conditions.

**Table 3:** The main effect of seasonal changes, locations, and planting methods on studied traits of sorghum at the three cuts as well as the mean and total of the three cuts under normal irrigation and drought conditions.

Factors	Normal conditions				Drought conditions			
	1st cut	2nd cut	3rd cut	Mean	1st cut	2nd cut	3rd cut	Mean
Plant height (cm)								
Growing seasons								
2023	164.08a	128.49a	124.62a	139.07a	152.54a	119.90a	112.11a	128.34a
2024	160.33b	129.96a	117.49b	136.08b	148.18b	111.36a	113.27a	124.97b
LSD 5%	3.60	NS	1.41	1.54	2.38	NS	NS	1.72
Locations								
Ras El Hekma	155.52b	124.22a	119.02b	132.92b	144.20b	116.41a	108.42b	123.23b
Wadi El-Raml	168.89a	134.49a	123.09a	142.23a	156.52a	114.85a	116.96a	130.08a
LSD 5%	3.60	NS	1.41	1.54	2.38	NS	2.01	1.72
Planting methods								
Hole farming	167.50a	137.43a	125.89a	143.61a	156.33a	123.33a	119.48a	133.76a
Row	164.34a	128.53a	121.66b	138.18b	149.00b	116.95a	112.43b	126.13b
Broad	154.78b	120.96b	115.62c	130.93c	145.75c	106.50a	106.17c	120.08c
LSD 5%	4.41	15.84	1.73	1.89	2.91	NS	2.47	2.11
	No. of Tillers\m2							
Growing seasons								
2023	52.56a	53.06a	39.09a	48.24a	46.72a	49.11a	33.78a	43.20a
2024	49.11b	48.50b	36.13b	44.58b	43.72b	44.67b	30.97b	39.79b
LSD 5%	3.07	2.13	1.94	1.28	2.42	2.16	0.89	1.27
Locations								
Ras El Hekma	52.11a	45.72b	36.06b	44.63b	47.39a	40.44b	30.72b	39.52b
Wadi El-Raml	49.56b	55.83a	39.17a	48.19a	43.06b	53.33a	34.03a	43.47a
LSD 5%	NS	2.13	1.94	1.28	2.42	2.16	0.89	1.27
Planting methods								
Hole farming	59.25a	59.33a	43.80a	54.13a	52.50a	54.92a	38.48a	48.63a
Row	50.50b	51.67b	37.31b	46.49b	45.67b	47.75b	32.73b	42.05b
Broad	42.75c	41.33c	31.73c	38.60c	37.50c	38.00c	25.92c	33.81c
LSD 5%	3.76	2.61	2.37	1.57	2.97	2.65	1.09	1.56

Factors	Normal conditions					Drought conditions				
	1st cut	2nd cut	3rd cut	Mean	Sum	1st cut	2nd cut	3rd cut	Mean	Sum
Fresh forage yield (t/fed)										
Growing seasons										
2023	16.57b	11.57a	6.82b	11.66b	34.97b	15.20b	10.43a	4.97b	10.20b	30.60b
2024	17.63a	9.98b	9.69a	12.43a	37.30a	16.33a	7.89b	8.65a	10.96a	32.88a
LSD 5%	0.87	0.41	0.45	0.30	0.91	1.01	0.54	0.48	0.44	1.33
Locations										
Ras El Hekma	16.13b	11.57a	6.62b	11.44b	34.32b	14.74b	10.12a	4.77b	9.88b	29.64b
Wadi El-Raml	18.08a	9.98b	9.90a	12.65a	37.96a	16.79a	8.20b	8.85a	11.28a	33.84a
LSD 5%	0.87	0.41	0.45	0.30	0.91	1.01	0.54	0.48	0.44	1.33
Planting methods										
Hole farming	19.43a	13.25a	9.28a	13.98a	41.95a	16.35a	10.42a	7.50a	11.42a	34.27a
Row	16.34b	11.02b	8.22b	11.86b	35.57b	15.63a	9.48b	6.73b	10.61b	31.84b
Broad	15.54b	8.07c	7.27c	10.29c	30.88c	15.32a	7.58c	6.21c	9.70c	29.11c
LSD 5%	1.07	0.50	0.55	0.37	1.11	NS	0.66	0.58	0.54	1.63
Dry forage yield (t/fed)										
Growing seasons										
2023	7.72b	5.38a	1.81b	4.97b	14.91b	6.32b	4.06a	1.23b	3.86b	11.38b
2024	8.19a	3.75b	3.74a	5.23a	15.68a	6.96a	2.70b	2.97a	4.21a	12.63a
LSD 5%	0.44	0.16	0.13	0.16	0.49	0.31	0.45	0.17	0.13	0.53
Locations										
Ras El Hekma	7.55b	5.23a	1.81b	4.86b	14.59b	6.13b	3.92a	1.20b	3.74b	11.04b
Wadi El-Raml	8.36a	3.90b	3.74a	5.33a	16.00a	7.15a	2.82b	3.00a	4.32a	12.97a
LSD 5%	0.44	0.16	0.13	0.16	0.49	0.31	0.45	0.17	0.13	0.53
Planting methods										
Hole farming	9.69a	5.57a	3.25a	6.17a	18.50a	7.11a	3.65a	2.49a	4.42a	13.25a
Row	7.44b	4.29b	2.64b	4.79b	14.37b	6.68b	3.34ab	2.13b	4.05b	12.15b
Broad	6.74c	3.85c	2.43c	4.34c	13.01c	6.12c	3.06b	1.68c	3.63c	10.61c
LSD 5%	0.54	0.19	0.16	0.20	0.60	0.38	0.56	0.21	0.16	0.65
Fresh water use efficiency (kg/m^3^)										
Growing seasons										
2023	7.53b	5.26a	3.10b	5.30b	15.89b	7.60b	5.21a	2.49b	5.10b	15.30b
2024	8.01a	4.54b	4.41a	5.65a	16.96a	8.17a	3.95b	4.33a	5.48a	16.44a
LSD 5%	0.40	0.19	0.20	0.14	0.41	0.50	0.27	0.24	0.22	0.67
Locations										
Ras El Hekma	7.33b	5.26a	3.01b	5.20b	15.60b	7.37b	5.06a	2.39b	4.94b	14.82b
Wadi El-Raml	8.22a	4.54b	4.50a	5.75a	17.25a	8.39a	4.10b	4.43a	5.64a	16.92a
LSD 5%	0.40	0.19	0.20	0.14	0.41	0.50	0.27	0.24	0.22	0.67
Planting methods										
Hole farming	8.83a	6.02a	4.22a	6.36a	19.07	8.18a	5.21a	3.75a	5.71a	17.13a
Row	7.43b	5.01b	3.74b	5.39b	16.17	7.81a	4.74b	3.37b	5.31b	15.92b
Broad	7.06b	3.67c	3.31c	4.68c	14.04	7.66a	3.79c	3.10c	4.85c	14.55c
LSD 5%	0.49	0.23	0.25	0.17	0.51	NS	0.33	0.29	0.27	0.82
Dry water use efficiency (kg/m3)										
Growing seasons										
2023	3.51b	2.45a	0.82b	2.26b	6.78b	3.16b	2.01a	0.62b	1.93b	5.79b
2024	3.72a	1.70b	1.70a	2.38a	7.13a	3.48a	1.35b	1.48a	2.10a	6.31a
LSD 5%	0.20	0.07	0.06	0.07	0.22	0.15	0.10	0.09	0.06	0.19
Locations										
Ras El Hekma	3.43b	2.38a	0.82b	2.21b	6.63b	3.07b	1.95a	0.60b	1.87b	5.62b
Wadi El-Raml	3.80a	1.77b	1.70a	2.42a	7.27a	3.57a	1.41b	1.50a	2.16a	6.49a
LSD 5%	0.20	0.07	0.06	0.07	0.22	0.15	0.10	0.09	0.06	0.19
Planting methods										
Hole farming	4.40a	2.53a	1.48a	2.80a	8.41a	3.56a	1.82a	1.25a	2.21a	6.63a
Row	3.38b	1.95b	1.20b	2.18b	6.53b	3.34b	1.67b	1.07b	2.03b	6.08b
Broad	3.06c	1.75c	1.10c	1.97c	5.91c	3.06c	1.55c	0.84c	1.82c	5.45c
LSD 5%	0.25	0.09	0.07	0.09	0.27	0.19	0.12	0.11	0.08	0.24

Means sharing different letters in the same column indicate statistically significant (*p* ≤ 0.05) differences according to the LSD test.

### The first-order interactions effects

The interaction between the 2024 season and the Wadi El-Raml location accounted for more positive effects of all studied traits at most cuts in both irrigation conditions than the other season and location interactions did (Table 4). The highest values of growth, forage yield, and water use efficiency (both fresh and dry) traits at all cuts were recorded in the Wadi El-Raml location at both seasons under both irrigation conditions, except for plant height at the second cut under drought conditions and for No. of Tillers/m^2^ at the first cut in both conditions. Wadi El-Raml location in the 2024 growing season increased plant height, forage yield, and water use efficiency (both fresh and dry) traits at all cuts in both irrigation conditions, except plant height at the third (normal) and second cuts (drought), forage yield, and water use efficiency (both fresh and dry) traits at the second cut in both conditions. While the Wadi El-Raml location in the 2023 growing season increased No. of Tillers\m^2^ at all cuts in both irrigation conditions, except at the first cut. Drought conditions reduced all studied traits at all cuts compared with the normal irrigation conditions according to the interaction between seasons and locations. Generally, our results indicated that increasing the forage yield and water use efficiency (both fresh and dry) of sorghum with the Wadi El-Raml location in both growing seasons under normal irrigation and drought conditions.

**Table 4:** Seasonal changes vs. locations for studied traits of sorghum at the three cuts as well as the mean and total of the three cuts under normal irrigation and drought conditions.

Seasons	Locations	Normal conditions				Drought conditions			
		1st cut	2nd cut	3rd cut	Mean	1st cut	2nd cut	3rd cut	Mean
Plant height (cm)									
2023	Ras El Hekma	159.90b	128.92	121.69b	136.84c	148.58b	120.00	111.56b	127.02b
	Wadi El-Raml	168.27a	128.07	127.56a	141.30b	156.50a	119.81	112.67b	129.66a
2024	Ras El Hekma	151.14c	119.51	116.36d	129.00d	139.82c	113.22	105.29c	119.44c
	Wadi El-Raml	169.51a	141.71	118.62c	143.15a	156.54a	109.28	121.24a	130.49a
LSD 5%	5.09	NS	2.00	2.18	3.36	NS	2.85	2.44	
No. of Tillers\m2									
2023	Ras El Hekma	53.89	47.67	37.11	46.22	49.11	42.00	32.22	41.11
	Wadi El-Raml	51.22	58.44	41.08	50.25	44.33	56.22	35.33	45.30
2024	Ras El Hekma	50.33	43.78	35.00	43.04	45.67	38.89	29.22	37.93
	Wadi El-Raml	47.89	53.22	37.26	46.12	41.78	50.44	32.72	41.65
LSD 5%	NS	NS	NS	NS	NS	NS	NS	NS	

Seasons	Locations	Normal conditions					Drought conditions				
		1st cut	2nd cut	3rd cut	Mean	Sum	1st cut	2nd cut	3rd cut	Mean	Sum
Fresh forage yield (t/fed)											
2023	Ras El Hekma	15.52	11.11b	6.38b	11.00	33.01	14.12	9.89bb	4.71b	9.57	28.72
	Wadi El-Raml	17.63	12.03a	7.27b	12.31	36.93	16.28	10.97a	5.23b	10.83	32.48
2024	Ras El Hekma	16.73	12.03a	6.86b	11.87	35.62	15.37	10.36ab	4.83b	10.19	30.56
	Wadi El-Raml	18.53	7.93c	12.53a	12.99	38.98	17.30	5.43c	12.47a	11.73	35.20
LSD 5%	NS	0.58	0.63	NS	NS	NS	0.76	0.67	NS	NS	
Dry forage yield (t/fed)											
2023	Ras El Hekma	7.30	5.03c	1.73b	4.69	14.07	5.93	3.76a	1.16b	3.60	10.43
	Wadi El-Raml	8.14	5.73a	1.88b	5.25	15.76	6.70	4.32a	1.31b	4.11	12.33
2024	Ras El Hekma	7.80	5.43b	1.88b	5.04	15.11	6.33	4.07a	1.24b	3.88	11.64
	Wadi El-Raml	8.58	2.07d	5.60a	5.42	16.25	7.59	1.33b	4.69a	4.54	13.61
LSD 5%	NS	0.22	0.18	NS	NS	NS	0.64	0.25	NS	NS	
Fresh water use efficiency (kg/m^3^)											
2023	Ras El Hekma	7.06	5.05b	2.90b	5.00	15.01	7.06	4.94b	2.36b	4.79	14.36
	Wadi El-Raml	8.01	5.47a	3.30a	5.59	16.78	8.14	5.48a	2.62b	5.41	16.24
2024	Ras El Hekma	7.61	5.47a	3.12ab	5.40	16.19	7.68	5.18ab	2.42b	5.09	15.28
	Wadi El-Raml	8.42	3.60c	5.69c	5.91	17.72	8.65	2.71c	6.24a	5.87	17.60
LSD 5%	NS	0.26	0.29	NS	NS	NS	0.38	0.34	NS	NS	
Dry water use efficiency (kg/m^3^)											
2023	Ras El Hekma	3.32	2.29b	0.79b	2.13	6.39	2.97	1.87b	0.58b	1.80	5.41
	Wadi El-Raml	3.70	2.61a	0.85b	2.39	7.16	3.35	2.16a	0.66b	2.06	6.17
2024	Ras El Hekma	3.55	2.47c	0.85b	2.29	6.87	3.17	2.03a	0.62b	1.94	5.82
	Wadi El-Raml	3.90	0.94d	2.55a	2.46	7.38	3.80	0.67c	2.34a	2.27	6.81
LSD 5%	NS	0.10	0.08	NS	NS	NS	0.14	0.12	NS	NS	

Means sharing different letters in the same column indicate statistically significant (*p* ≤ 0.05) differences according to the LSD test.

Regarding the interaction between seasons and planting methods, the hole farming method showed the highest values for growth, forage yield, and water use efficiency (both fresh and dry) traits in both growing seasons at all cuts under normal irrigation and drought conditions. This was followed by the drill-in-row method and the broadcasting method, as shown in Table 5. Except for the third cut and the average of the three cuts, the hole farming approach produced the highest plant height and number of tillers/m^2^ attributes over the 2023 growing season at all cuts under both irrigation regimes. While, except for the second cut, the hole farming method produced the highest forage yield and water use efficiency (both fresh and dry) traits during the 2024 growing season at all cuts under both irrigation regimes. Drought conditions reduced all studied traits at all cuts compared with the normal irrigation conditions according to the interaction between seasons and planting methods. Generally, our results indicated that increasing the forage yield and water use efficiency (both fresh and dry) of sorghum with the hole farming method in both growing seasons under normal irrigation and drought conditions.

**Table 5:** Seasonal changes vs. planting methods for studied traits of sorghum at the three cuts as well as the mean and total of the three cuts under normal irrigation and drought conditions.

Seasons	Planting methods	Normal conditions				Drought conditions			
		1^st^ cut	2^nd^ cut	3^rd^ cut	Mean	1^st^ cut	2^nd^ cut	3^rd^ cut	Mean
Plant height (cm)									
2023	Hole farming	168.17	139.60	127.03a	144.93	157.93	124.18	115.75b	132.62a
	Row	167.03	127.02	125.10a	139.72	151.12	120.07	111.97c	127.72b
	Broad	157.05	118.87	121.73b	132.55	148.57	114.56	108.62c	124.68c
2024	Hole farming	166.83	135.27	124.75a	142.28	154.73	122.30	123.20a	134.89a
	Row	161.65	130.05	118.22c	136.64	146.88	113.83	112.88bc	124.53c
	Broad	152.50	123.48	109.50d	129.31	142.93	99.78	103.72d	115.48d
LSD 5%	NS	NS	2.45	NS	NS	NS	3.49	2.98	
No. of Tillers\m2									
2023	Hole farming	61.17	62.50	45.17	56.28	54.50	57.83	39.67	50.67
	Row	52.17	53.83	38.72	48.24	46.83	50.17	34.50	43.83
	Broad	44.33	42.83	33.40	40.19	38.83	39.33	27.17	35.11
2024	Hole farming	57.33	56.17	42.43	51.98	50.50	52.00	37.28	46.59
	Row	48.83	49.50	35.90	44.74	44.50	45.33	30.97	40.27
	Broad	41.17	39.83	30.05	37.02	36.17	36.67	24.67	32.50
LSD 5%	NS	NS	NS	NS	NS	NS	NS	NS	

Seasons	Planting methods	Normal conditions					Drought conditions				
		1^st^ cut	2^nd^ cut	3^rd^ cut	Mean	Sum	1^st^ cut	2^nd^ cut	3^rd^ cut	Mean	Sum
Fresh forage yield (t/fed)											
2023	Hole farming	18.91	14.30	7.53	13.58	40.74	15.62	11.82	5.67	11.03	33.10
	Row	15.89	11.75	7.02	11.55	34.65	15.30	11.00	4.90	10.40	31.20
	Broad	14.93	8.67	5.92	9.84	29.52	14.69	8.47	4.35	9.17	27.50
2024	Hole farming	19.96	12.19	11.02	14.39	43.17	17.09	9.02	9.33	11.81	35.43
	Row	16.79	10.28	9.43	12.16	36.49	15.96	7.96	8.56	10.83	32.48
	Broad	16.15	7.47	8.63	10.75	32.25	15.96	6.70	8.07	10.24	30.72
LSD 5%	NS	NS	NS	NS	NS	NS	NS	NS	NS	NS	
Dry forage yield (t/fed)											
2023	Hole farming	9.41	6.55a	2.01d	5.99	17.97	6.54bc	4.39	1.39d	4.10b	12.31
	Row	7.22	5.10b	1.78e	4.70	14.10	6.31cd	3.95	1.25de	3.84c	11.51
	Broad	6.54	4.50c	1.64e	4.22	12.67	6.11d	3.79	1.06e	3.63c	10.32
2024	Hole farming	9.96	4.59c	4.48a	6.34	19.03	7.69a	2.91	3.60a	4.73a	14.19
	Row	7.66	3.48d	3.51b	4.88	14.65	7.06b	2.74	3.01b	4.27b	12.80
	Broad	6.94	3.19e	3.22c	4.45	13.36	6.14cd	2.46	2.29c	3.63c	10.90
LSD 5%	NS	0.27	0.23	NS	NS	0.53	NS	0.30	0.22	NS	
Fresh water use efficiency (kg/m^3^)											
2023	Hole farming	8.59	6.50	3.42	6.17	18.52	7.81	5.91	2.84	5.52	16.55
	Row	7.22	5.34	3.19	5.25	15.75	7.65	5.50	2.45	5.20	15.60
	Broad	6.79	3.94	2.69	4.47	13.42	7.34	4.23	2.17	4.58	13.75
2024	Hole farming	9.07	5.54	5.01	6.54	19.62	8.54	4.51	4.67	5.91	17.72
	Row	7.63	4.67	4.28	5.53	16.59	7.98	3.98	4.28	5.41	16.24
	Broad	7.34	3.39	3.92	4.89	14.66	7.98	3.35	4.03	5.12	15.36
LSD 5%	NS	NS	NS	NS	NS	NS	NS	NS	NS	NS	
Dry water use efficiency (kg/m^3^)											
2023	Hole farming	4.28	2.98a	0.91d	2.72	8.17	3.27ab	2.19	0.70d	2.05b	6.16b
	Row	3.28	2.32b	0.81e	2.14	6.41	3.15b	1.98	0.63de	1.92c	5.76c
	Broad	2.97	2.05c	0.74e	1.92	5.76	3.05b	1.87	0.53e	1.82c	5.45c
2024	Hole farming	4.53	2.08c	2.04a	2.88	8.65	3.84c	1.45	1.80a	2.36a	7.09a
	Row	3.48	1.58d	1.60b	2.22	6.66	3.53a	1.37	1.50b	2.13b	6.40b
	Broad	3.16	1.45e	1.46c	2.02	6.07	3.07b	1.23	1.15c	1.82c	5.45c
LSD 5%	NS	0.12	0.10	NS	NS	0.27	NS	0.15	0.11	0.34	

Means sharing different letters in the same column indicate statistically significant (*p* ≤ 0.05) differences according to the LSD test.

Regarding the relationship between locations and planting methods (Table 6), an Wadi El-Raml location at all cuts under both irrigation conditions produced the greatest outcomes for growth, forage yield, and water use efficiency (both fresh and dry) traits of sorghum across the three planting methods under study. Additionally, under normal irrigation and drought conditions, the hole farming approach yielded the greatest values for all examined traits, followed by the drill-in-row method and the broadcasting method at all cuttings in both locations. Compared with other interactions of locations and planting methods, the maximum values for all studied traits were found by the hole farming method across the Wadi El-Raml location at all cuts in both irrigation conditions, except for plant height at the second cut in drought conditions, for No. of Tillers\m2 at the first cut in both conditions, for forage yield, and water use efficiency (both fresh and dry) traits at the second cut in both conditions. Drought conditions reduced all studied traits at all cuts compared with the normal irrigation conditions according to the interaction between locations and planting methods. Every first-order interaction showed a range of patterns. However, statistical analysis revealed that for the majority of the features under investigation at all cuts, the 2024 growing season’s hole farming method with Wadi El-Raml location yielded the greatest values for these traits.

**Table 6:** Locations vs. planting methods studied traits of sorghum at the three cuts as well as the mean and total of the three cuts under normal irrigation and drought conditions.

Locations	Planting methods	Normal conditions				Drought conditions			
		1^st^ cut	2^nd^ cut	3^rd^ cut	Mean	1^st^ cut	2^nd^ cut	3^rd^ cut	Mean
Plant height (cm)									
Ras El Hekma	Hole farming	160.28	135.60	123.60	139.83	150.32	123.67	114.85	129.61
	Row	157.83	121.92	119.72	133.16	143.02	117.33	107.92	122.76
	Broad	148.45	115.13	113.75	125.78	139.27	106.60	102.50	117.33
Wadi El-Raml	Hole farming	174.72	139.27	128.18	147.39	162.35	122.92	124.10	137.91
	Row	170.85	135.15	123.60	143.20	154.98	116.57	116.93	129.49
	Broad	161.10	127.96	117.48	136.09	152.23	106.42	109.83	122.83
LSD 5%	NS	NS	NS	NS	NS	NS	NS	NS	
No. of Tillers\m2									
Ras El Hekma	Hole farming	60.67	52.83c	41.50	51.67	55.33	46.00c	37.33	46.22
	Row	53.33	45.33d	36.33	45.00	46.83	41.33d	31.00	39.72
	Broad	42.33	39.00e	30.33	37.22	40.00	34.00e	23.83	32.61
Wadi El-Raml	Hole farming	57.83	65.83a	46.10	56.59	49.67	63.83a	39.62	51.04
	Row	47.67	58.00b	38.28	47.98	44.50	54.17b	34.47	44.38
	Broad	43.17	43.67d	33.12	39.98	35.00	42.00d	28.00	35.00
LSD 5%	NS	3.70	NS	NS	NS	3.74	NS	NS	

Locations	Planting methods	Normal conditions					Drought conditions				
		1^st^ cut	2^nd^ cut	3^rd^ cut	Mean	Sum	1^st^ cut	2^nd^ cut	3^rd^ cut	Mean	Sum
Fresh forage yield (t/fed)											
Ras El Hekma	Hole farming	18.42	14.10	7.28d	13.27	39.80	15.22	11.72	5.53	10.82	32.47
	Row	15.48	11.88	6.82d	11.39	34.18	14.87	10.40	4.60	9.96	29.87
	Broad	14.48	8.73	5.75e	9.66	28.97	14.15	8.25	4.18	8.86	26.58
Wadi El-Raml	Hole farming	20.45	12.39	11.27a	14.70	44.11	17.49	9.12	9.47	12.02	36.07
	Row	17.19	10.15	9.63b	12.32	36.96	16.39	8.56	8.86	11.27	33.82
	Broad	16.60	7.40	8.80c	10.93	32.79	16.49	6.92	8.23	10.54	31.63
LSD 5%	NS	NS	0.77	NS	NS	NS	NS	NS	NS	NS	
Dry forage yield (t/fed)											
Ras El Hekma	Hole farming	9.20	6.40a	2.00d	5.87	17.60	6.30bc	4.20	1.35d	3.95c	11.85c
	Row	7.05	4.95b	1.75e	4.58	13.75	6.15cd	3.90	1.23de	3.76c	11.28c
	Broad	6.40	4.35c	1.67e	4.14	12.42	5.95d	3.62	1.02e	3.52d	9.98d
Wadi El-Raml	Hole farming	10.17	4.74b	4.49a	6.47	19.40	7.92a	3.09	3.64a	4.88a	14.65a
	Row	7.83	3.63d	3.54b	5.00	15.00	7.21b	2.79	3.03b	4.34b	13.03b
	Broad	7.08	3.34e	3.19c	4.54	13.61	6.30cd	2.60	2.34c	3.75c	11.24c
LSD 5%	NS	0.27	0.23	NS	NS	0.53	NS	0.30	0.22	0.92	
Fresh water use efficiency (kg/m^3^)											
Ras El Hekma	Hole farming	8.37	6.41	3.31d	6.03	18.09	7.61	5.86	2.77	5.41	16.23
	Row	7.04	5.40	3.10d	5.18	15.54	7.43	5.20	2.30	4.98	14.93
	Broad	6.58	3.97	2.61e	4.39	13.17	7.08	4.13	2.09	4.43	13.29
Wadi El-Raml	Hole farming	9.29	5.63	5.12a	6.68	20.05	8.74	4.56	4.74	6.01	18.04
	Row	7.81	4.61	4.38b	5.60	16.80	8.20	4.28	4.43	5.64	16.91
	Broad	7.54	3.36	4.00c	4.97	14.91	8.25	3.46	4.11	5.27	15.82
LSD 5%	NS	NS	0.35	NS	NS	NS	NS	NS	NS	NS	
Dry water use efficiency (kg/m^3^)											
Ras El Hekma	Hole farming	4.18	2.91a	0.91d	2.67	8.00	3.15c	2.10	0.68d	1.98c	5.93c
	Row	3.20	2.25b	0.80e	2.08	6.25	3.08c	1.95	0.62de	1.88c	5.64c
	Broad	2.91	1.98c	0.76e	1.88	5.64	2.98c	1.80	0.51e	1.76d	5.28d
Wadi El-Raml	Hole farming	4.62	2.15b	2.04a	2.94	8.82	3.96a	1.55	1.82a	2.44a	7.33a
	Row	3.56	1.65d	1.61b	2.27	6.82	3.61b	1.39	1.51b	2.17b	6.51b
	Broad	3.22	1.52e	1.45c	2.06	6.19	3.15c	1.30	1.17c	1.87c	5.62c
LSD 5%	NS	0.12	0.10	NS	NS	0.27	NS	0.15	0.11	0.34	

Means sharing different letters in the same column indicate statistically significant (*p* ≤ 0.05) differences according to the LSD test.

### Stress tolerance index (STI)

**Fig. 2:**
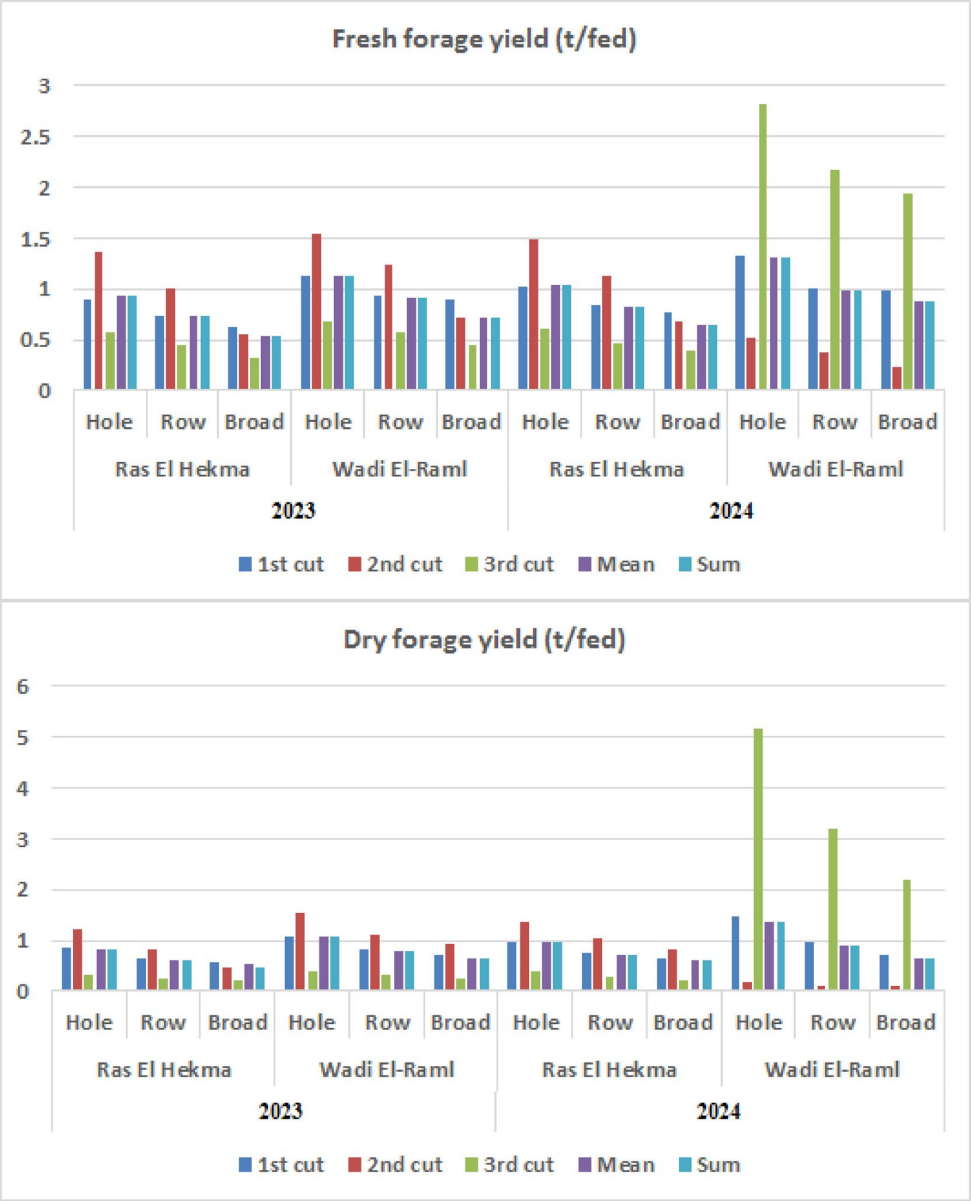
Stress tolerance index for fresh and dry forage yields (t/fed) traits at all cuts as affected by the seasons, locations, and planting methods.

The STI of fresh and dry forage yields (t/fed) traits of sorghum plants at all cuts affected by the seasons, locations, and planting methods are given in Fig. 2. In comparison to alternative planting methods, the sorghum plants grown by the hole farming approach showed greater STI values at all cuttings throughout all seasons and locales for both fresh and dry fodder yields. Under the planting practices and growing seasons, the STI rose for fresh and dried forage yields at all cuts in Wadi El-Raml locations when compared to the Ras El Hekma location. Generally, the STI was highest for the sorghum plants grown using the hole farming method at the Wadi El-Raml location in both growing seasons.

### Principal component analysis (PCA)

The PCs formed were equal to the number of traits under study, but the four PCs accounted for 100% of the total variation among traits under experimental factors at the mean of the three cuts under normal irrigation and drought conditions, as shown in Table 7. PCA indicated PC1 extracted 90.50% of the total variation among the studied variables with an eigenvalue > 1 under both irrigation conditions. While the other three PCs had eigenvalues < 1. The largest contribution to the overall variation in this study was PC1, which was followed by PC2 (6.22%), PC3 (2.85%), and PC4 (0.43%). PC1 and PC2 extract 96.72%. PC1 and PC2 contributed to all studied traits at the mean of the three cuts with values ranging from 0.28 to 0.30 and from − 0.37 to 0.44, respectively. Also, PC1 exhibited a positive correlation with the hole farming method (1.60) at Wadi El-Raml location (0.74) in the 2024 growing season (0.18). PC2 is associated with the hole farming method (0.59) at Ras El Hekma location (0.77) in the 2023 growing season (1.47).

**Fig. 3:**
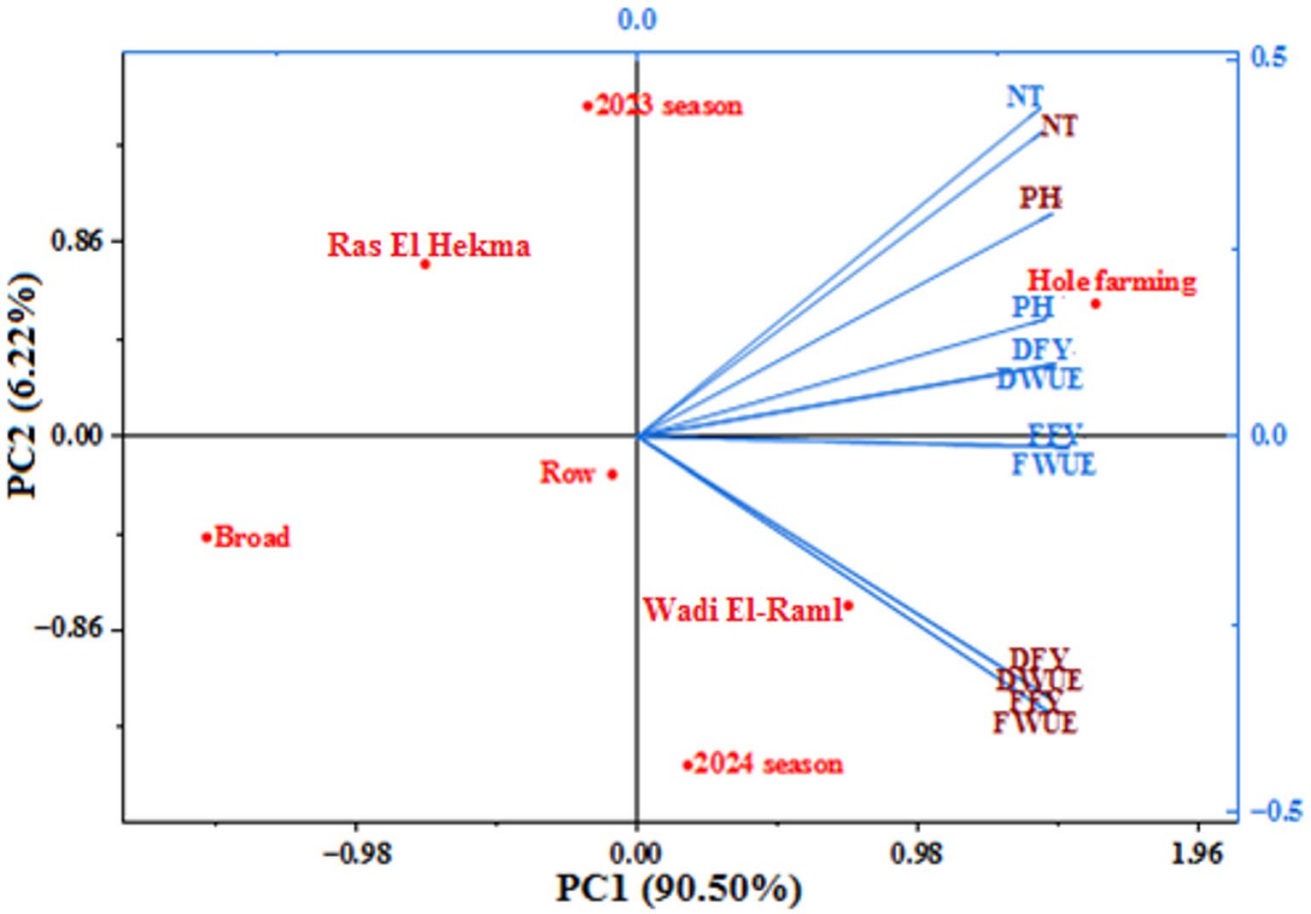
The relationships between the studied traits at the mean of the three cuts across the growing seasons, locations, and planting methods under normal (blue color) and drought (brown color) conditions using the biplot diagram between PC1 and PC2.

PC1 and PC2 for the experimental factors and all studied traits at the mean of the three cuts under normal irrigation and drought conditions are displayed in Fig. 3. Every trait examined showed variability as a result of changes in planting methods at locations throughout the course of the growing years, as illustrated by the biplot diagram. All traits exhibited a steep angle between them and showed a positive correlation with experimental factors at the mean of the three cuts under both irrigation conditions, as indicated by the biplot diagram between PC1 and PC2. A perfect positive correlation (angle = 0) was noticed between fresh forage yield and fresh water use efficiency traits, between dry forage yield and dry water use efficiency traits under normal conditions, as well as between fresh forage yield and dry forage yield, and between fresh water use efficiency and dry water use efficiency under drought conditions. Also, all other possible pairs among all studied traits showed a positive correlation in both irrigation conditions.

According to the first two components obtained using PCA, Fig. 3 shows the distribution of experimental factors along different ordinate axes as well as the distribution of different traits among the experimental factors under both normal irrigation and drought conditions. The importance of these characteristics for the corresponding experimental factors is indicated by the experimental factors’ placement along each vector. The hole farming method (first quarter) with Wadi El-Raml locations and the 2024 growing season (fourth quarter) displayed importance for forage yield and water use efficiency traits in both irrigation conditions, which were positively related to PC1. In general, the dry forage yield and dry water usage efficiency of sorghum under normal irrigation conditions were close to the hole farming approach.

**Table 7:** Results of PCA in the first four PCs for the studied traits of Pearl millet at the mean of the three cuts as affected by the three experimental factors.

Irrigations	Traits	PC1	PC2	PC3	PC4
Normal	Plant height (PH)	0.28	0.16	0.55	0.33
	Number of tillers/m2 (NT)	0.28	0.44	0.06	-0.32
	Fresh forage yield (FFY)	0.30	-0.01	-0.25	-0.26
	Dry forage yield (DFY)	0.29	0.10	-0.46	0.37
	Fresh water use efficiency (FWUE)	0.30	-0.01	-0.26	-0.25
	Dry water use efficiency (DWUE)	0.29	0.10	-0.46	0.33
Drought	Plant height (PH)	0.29	0.30	0.26	0.43
	Number of tillers/m2 (NT)	0.28	0.40	0.15	-0.44
	Fresh forage yield (FFY)	0.29	-0.37	0.14	0.02
	Dry forage yield (DFY)	0.29	-0.35	0.08	-0.11
	Fresh water use efficiency (FWUE)	0.29	-0.37	0.14	0.00
	Dry water use efficiency (DWUE)	0.29	-0.35	0.10	-0.11
2023 growing season		-0.17	1.47	0.79	0.45
2024 growing season		0.18	-1.46	-0.82	-0.40
Ras El Hekma		-0.74	0.77	-1.10	-0.81
Wadi El-Raml		0.74	-0.75	1.12	0.79
Hole farming		1.60	0.59	-0.95	0.40
Row		-0.09	-0.17	1.13	-1.65
Broadcasting		-1.51	-0.45	-0.17	1.21
Eigenvalues		10.86	0.75	0.34	0.05
Variance (%)		90.50	6.22	2.85	0.43
Cumulative (%)		90.50	96.72	99.57	100.00

## Discussion

In the Mediterranean, where the consequences of climate change are evident, agricultural production is significantly impacted. Recent models indicate greater temperatures and less precipitation, with the trend likely to be particularly pronounced during the years hottest times^38^. One of the main factors restricting crop growth and productivity is drought. With a focus on creating cultivars that can withstand drought, improving drought resistance is a crucial tactic to maintain increased yields^17^. In this study, the Sorghum Hendy variety was selected as experimental material to evaluate the growth, forage yield, and water use efficiency (in fresh and dry) in two locations, Matrouh Governorate, Egypt, using three planting techniques in each of the growing years 2023 and 2024 under normal irrigation and drought conditions.

Based on the ANOVA test, growth, forage yield, and water use efficiency (in fresh and dry) at most cuts were affected significantly by seasons, locations, and planting methods under normal irrigation and drought conditions. The year had a substantial impact on the yield of dry forage^39^, green fodder, and dry matter^13^. Ertekin and Yılmaz^40^ found no significant effect of year on fresh forage yield or dry matter yield. These results indicate a significant effect of location on fodder yield, which varied with the location of evaluation^41^. Locations differed significantly in terms of fresh and dry forage yield, plant height at the two cuts and three cuts, and total sorghum yield^42^ and on plant height and forage yield of sorghum^43^. Planting methods significantly affect growth, yield, and quality of forage crops^44^, fresh forage yield and dry matter yield^40^, plant height, number of tillers/m^2^, yield of fresh and dry forage at each cut, as well as the total yield of fresh and dry forage in the first and second summer seasons^45^, and biomass yield of sorghum^28^. These results suggest that there is diversity among the experimental elements being studied, which suggests that sorghum’s water use efficiency and fodder productivity at all cuts can be improved under drought conditions. According to Dolapčev Rakić et al.^13^, the variations in growing seasons are a reflection of the impact of different climatic circumstances and increase the characteristics’ reliance on environmental factors. The results of Erdurmus et al.^43^, and Ertekin and Yılmaz^40^, Mekasha et al.^46^ and Rady et al.^42^, on growth, fresh and dry forage yields at the cuts, and total forage yield were comparable to the data gathered from the interaction between experimental factors for the current study. This response might possibly have been influenced by the growing season’s environmental circumstances^47^. The results of CV% would imply that the experimental factors under investigation differ significantly from one another for forage yield and water use efficiency of sorghum at all cuts under drought conditions. Plant height (11.11% and 10.43%), green fodder yield (31.25% and 38.11%), and dry matter yield (41.06% and 51.81%) all showed high CV% values in both growth seasons, according to Dolapčev Rakić et al.^13^.

Our results showed that drought stress conditions reduced all examined traits at all cuts in both seasons and locations compared with the normal irrigation conditions. Sorghum plant height and fodder productivity were gradually reduced by drought stress^48^. According to Hussein et al.^49^, irrigated fodder may benefit from a moderate amount of inadequate irrigation as a water management approach, particularly in regions with limited water supplies. Consequently, a more effective and sustainable method of water use may be achieved by irrigating a greater area with the water saved by deficit irrigation. All of the traits that were analyzed at the first cut outperformed the second and third cuts under all planting methods in all locations and seasons.

Compared to the 2023 growing season, the 2024 growing season saw increases in fresh forage yield, dry forage yield, fresh water use efficiency, and dry water use efficiency at the sum of the three cuttings of 3.59%, 5.21%, 3.59%, and 4.30% under drought conditions, respectively. In contrast, the 2023 growing season produced the maximum plant height and number of tillers/m^2^ at all cuts under both conditions. These findings concur with those of Dolapčev Rakić et al.^13^, who found that, possibly as a result of improved environmental circumstances, one of the growing seasons was generally more favorable for biomass and yield features such as plant height (0.85%), green fodder production (8.28%), and dry matter yield (12.14%). Compared to grain sorghum, forage sorghum has a higher base temperature, more leaf area, and a stronger tolerance to heat and water stressors^50^. Temperature affects yields because greater mineralization rates in warmer climates increase the nutrient’s availability^51^. Our findings are consistent with those of Druille et al.^50^ and Pembleton et al.^52^, who found that raising the mean annual temperature scenario without altering the yearly precipitation increased the yield of feed sorghum. All studied traits at most cuts raised in the Wadi El-Raml location more than in Ras El Hekma location in both irrigation conditions. At the sum of the three cuttings, the Wadi El-Raml location experienced improvements in fresh forage yield (6.62%), dry forage yield (8.04%), fresh water use efficiency (6.56%), and dry water use efficiency (7.18%) under drought conditions, in comparison to the Ras El Hekma location. The yield of forage sorghum is greatly influenced by the site of its cultivation. Plant development, maturity, and nutritional value can be influenced by a variety of factors, including soil type, altitude, and climate (temperature, rainfall, and sunlight). Additionally, different places may have differing degrees of disease and insect pressure, which could affect the total productivity of forage sorghum.

Under normal irrigation and drought conditions, the hole farming method improved all the traits under study at all cuttings when compared to alternative planting methods. Under drought conditions, the hole farming method outperformed the drill-in-row and broadcasting in terms of fresh forage yield (2.55% and 5.42%), dry forage yield (3.05% and 7.33%), fresh water use efficiency (2.54% and 5.42%), and dry water use efficiency (3.03% and 6.50%) at the total of the three cuttings, respectively. Plant height is significantly influenced by planting methods. Reduced plant density, improved light penetration, and efficient soil moisture and nutrient usage are the reasons given for the increased growth with pit techniques^53^. Our results are consistent with those of Chattha et al.^24^, Haggag et al.^54^, and Hssan et al.^55^, who found that the broadcasting method produced the lowest fresh forage yields, while the hills/ridge seeding method produced the highest. Additionally, EL-Gaafarey et al.^45^ found that, when compared to broadcasting on the top of the rows, planting in hills on top of the rows resulted in the greatest significant increase in plant height, number of tillers/m^2^, and yield of fresh and dry forage at each cut as well as the total yield of fresh and dry forage (ton fed^-1^) in both seasons. Plant growth, which was influenced by competition between plants for nutrients, moisture, sunlight, and other growth factors, was more favorable when planting in hills on top of the rows, which may account for this rise^45^. In comparison to the traditional flatbed planting approach, Wondimu et al.^56^ discovered a 15–24% increase in seasonal soil moisture content with tied and open ridges.

By comparing the means under normal and drought stress circumstances, the STI is utilized to identify high-tolerance genotypes^37^. The sorghum plants cultivated at the Wadi El-Raml location using the hole farming method in both growing seasons reported the highest STI for fresh and dry forage yields at all cuts in both seasons and locations when compared to all other experimental factors in our study. As a result, the forage yield at all cuts of sorghum plants grown using the hole farming method was the least vulnerable to drought stress. Sorghum genotypes that fared well under stress treatment were classified as high-tolerant, and those that fared poorly were classified as sensitive, according to STI value^57^.

Combining the techniques of PCA and correlation allows for the identification of significant factors influencing folder yield and water use efficiency. PC1 and PC2 account for almost 96.72% of the total variance in all the variables analyzed under the seasons, locations, and planting methods at the mean of the three cuts under normal and drought conditions. Consequently, the results of PC1 and PC2 can be utilized to summarize the original variables in any further data analysis, as well as to explain the overall variance and the PC collection. Nearly 90.50% of the variability in the measured data for the original variables was explained by PC1, with eigenvalues higher than one. A substantial amount of the variability is present in the first principal component, while the second and subsequent components show less variability^58^. According to Hair et al.^59^, PCs were deemed significant and valuable if their component loadings were more than ± 0.3 and their eigenvalues were greater than unity. These findings suggested that PC1 was influenced by the hole farming method for forage yield and water use efficiency of sorghum in both irrigation conditions. An estimate of the correlation between the trait vectors is provided by the cosine of the angle between them in the PCA biplot^60^. The approximate angles of the vectors and the contribution of the same trait pairs in the PCA biplot roughly match the correlations between trait pairs^61^. In both irrigation conditions, every pair of forage yield and water use efficiency (in fresh and dry) that could be found exhibited a positive connection. According to these findings, choosing these characteristics would help boost the potential fodder production and water use efficiency of sorghum. Positive correlations were found between plant height and number of tillers/m^2^^62^ and between forage yield and plant height under stress^63,64^.

The significance of the characteristics under investigation as the primary contributing traits for sorghum’s forage yield and water use efficiency during drought conditions was shown using PCA based on the phenotypic correlation. In general, the Sorghum Hendy variety grown using the hole farming method with the Wadi El-Raml location in both growing seasons provided the highest forage yield and water use efficiency (in fresh and dry) of sorghum in the Matrouh Governorate, Egypt, according to the statistical analysis of the relationship between the variables examined.

## Conclusions

Different growing seasons, locations, and planting methods significantly impact growth, forage yield, and water use efficiency in both fresh and dry at most cuts under normal and drought conditions. Raised the hole farming method generally produces the highest forage yield and water use efficiency at total cuts, followed by the row method, while broadcasting results in the minimum values. The highest forage yield and water use efficiency (in both fresh and dry) at all cuts were obtained from hole farming method in the Wadi El-Raml location under both irrigation conditions. Therefore, the hole farming method came to the fore in terms of forage yield and water use efficiency and is more effective under drought stress conditions, which can be recommended to achieve higher forage productivity of the Sorghum Hendy variety when water is scarce in Matrouh Governorate, Egypt.

Future research should focus on identifying and implementing strategies to increase exploitable yield in water-limited cropping regions.

## Data Availability

The data are presented within the manuscript as tables and figures.
